# The Pearl in the Oyster

**Published:** 1883-09

**Authors:** 


### THE PEARL IN THE OYSTER.

Sometimes the most useful suggestions and ingenious contrivances are obtained from the most unlikely sources. A sharply observant dentist chanced not long since to stray into the office of a confrere who was located in a country hamlet, and he confessed himself astonished at the originality and fertility of resource observable in his self-taught acquaintance. The man was filling a tooth, and was using a piece of rubber-dam that was old and rotten. Into two or three holes through it he had stuffed some cotton dipped in a solution of gum-sandarach, which had effectually stopped them. But as there were yet leaks, he had rolled up half a sheet of bibulous paper, stuck one end down between the teeth, and that was acting as a capital siphon to draw off the saliva.

Not having a patent air-syringe he was using a common chip-blower, the nozzle of which he held near and just above the flame of his alcohol lamp, and thus he obtained heated air. "Everything in the office," said Dr. Frank Darby, the visitor, "evinced the same originality of contrivance in overcoming his lack of modern conveniences, and notwithstanding the absence of many instruments that are considered essential to successful practice, he was, through innate mechanical genius, doing excellent work."

